# Fungal Community, Metabolic Diversity, and Glomalin-Related Soil Proteins (GRSP) Content in Soil Contaminated With Crude Oil After Long-Term Natural Bioremediation

**DOI:** 10.3389/fmicb.2020.572314

**Published:** 2020-09-17

**Authors:** Anna Gałązka, Jarosław Grządziel, Rafał Gałązka, Karolina Gawryjołek, Aleksandra Ukalska-Jaruga, Bozena Smreczak

**Affiliations:** ^1^Department of Agriculture Microbiology, Institute of Soil Science and Plant Cultivation – State Research Institute, Puławy, Poland; ^2^Department of Soil Science Erosion and Land Protection, Institute of Soil Science and Plant Cultivation – State Research Institute, Puławy, Poland

**Keywords:** fungal community, genetic diversity, metabolic profiles, glomalin-related soil proteins, Biolog FFPlates, ITS gene region, NGS

## Abstract

Fungi have increased tolerance to environmental stress (also related to the access of pollutants, e.g., trace elements and polycyclic aromatic hydrocarbons PAHs). The aim of the study was to evaluate the mycobiome and functional diversity of fungi in long-term crude-oil contaminated soils as the potential bioremediators of oil contaminated sites. Samples were taken from three historical oil wells (over a century old) at two distances: within a 0.5 m radius of the oil wells (OWP1, OWP2, and OWP3) and within a 3 m radius from the oil wells as the controls (OW1, OW2, and OW3). Next generation sequencing (for the ITS region) was accompanied with determination of the functional fungal community based on Biolog FFPlates, glomalin related soil protein (GRSP) content, trace element and PAHs concentration. The research hypothesis assumed that long-term natural bioremediation of crude oil contaminated soils can contribute to intensive development of a unique fungal community adapted to the contamination conditions. The identification of such fungi can be of particular importance in soil bioremediation. There were significant differences in the fungal community and functional diversity between the soil samples. The soils collected directly from the oil wells were characterized by higher biological activity and higher diversity of PAH-degrading fungal candidates compared to the soils collected within 3 m of the oil wells. The total glomalin-related soil proteins (T-GRSP) and easily-extractable glomalin-related soil proteins (EE-GRSP) contents were lower in soil samples taken directly from the crude oil well. The control soil (OW) subjected to a long-term natural remediation may already have sufficient conditions for the growth and development of mycorrhizal fungi. The mycobiome of the soils collected directly from the oil wells (OWP1, OWP2, and OWP3) was characterized by a 35% share of PAH-degrading candidates, compared to the soil collected at the 3 m distance from the oil wells (OW1, OW2, and OW3) at < 5%. The main PAH-degrading fungal candidates belong to genera *Ilyonectria*, *Chaetomium*, *Gibberella*, *Paraphoma*, *Schizothecium*, *Pseudorobillarda*, *Tetracladium*, *Ganoderma*, *Cadophora*, *Exophiala*, *Knufia*, *Mycoleptodiscus*, *Cyphellophora*, *Fusicolla*, *Devriesia*, *Didymella*, *Plenodomus*, *Pyrenochaetopsis*, *Symbiotaphrina*, *Phallus*, *Coprinellus*, *Plectosphaerella*, *Septoriella*, and *Hypholoma*. The share of three- and four-ringed PAHs in soil was higher as the distance from the oil well increased. These results may indicate that more effective degradation processes occur closer to the oil wells.

## Introduction

The constantly increasing pollution of soils, air and waters associated with industrial activity, low-efficiency metal extraction methods, agriculture chemisation and/or problems related to oil extraction is a severe threat to health (Andreoni and Gianfreda, 2007; Ciesielczuk et al., 2014). The biological decomposition of organic pollutants, including petroleum-derived substances and polycyclic aromatic hydrocarbons (PAHs), by microorganisms (bacteria and fungi) is one of the most effective methods for removal of these compounds from the soil environment (Liang et al., 2011; Gałązka and Grządziel, 2016). Bioremediation use live microorganisms to catalyze the decomposition or transformation of pollutants. In comparison with other organisms, microorganisms exhibit a unique ability to adapt to extreme environmental conditions (Li et al., 2008; Hossain et al., 2017). Both bacteria and fungi are involved in the process of natural bioremediation of contaminated soils (Andreoni and Gianfreda, 2007). The metabolic activity of microorganisms that inhabit contaminated soil results in total or partial transformation of petroleum products into stable non-toxic products in the process of natural bioremediation (Wolińska et al., 2018). These products are CO_2_ and H_2_O in aerobic conditions and CH_4_ in an anaerobic environment. Aerobic remediation of organic pollutants in the soil is a more effective process. Biological degradation of petroleum substances can be achieved *via* basic bioremediation, i.e., monitoring processes that occur naturally at the contamination site, biostimulation (stimulation of autochthonous microorganisms to accelerate the biodegradation process) and bioaugmentation (introduction of microorganisms to enhance biodegradation at the contaminated site) (Andreoni and Gianfreda, 2007).

Monitoring naturally occurring bioremediation processes at the contamination site is highly important due to the possibility of recruiting autochthonous bacterial and fungal strains capable of decomposing pollutants (Gałązka et al., 2018). Mixed microbial cultures with diverse physiological characteristics can decompose complex mixtures such as crude oil (Baldrian, 2003; Huhe et al., 2017). Less than 1% of the total number of soil bacteria and fungi can decompose hydrocarbons. The number of heterotrophic fungi capable of hydrocarbon utilization as a sole carbon and energy source varies among ecosystems (soil, air and water). Petroleum can be decomposed by mixed microbial cultures characterized by varied activity and ability to utilize hydrocarbons as a carbon and energy source (Bundy et al., 2009). Co-metabolic hydrocarbon degradation plays a crucial role in the bioremediation process. In this case, degraded hydrocarbons are co-substrates rather than a carbon and energy source. Most frequently, hydrocarbon degradation proceeds sequentially with the involvement of various microbial groups that cooperate with each other (Gałązka and Gałązka, 2015).

Natural bioremediation of soil contaminated of crude oil is often associated with the development of natural flora in the affected area. Most often in this process is involved rhizosphere of meadow plants, similarly to the oil well (OW) rhizosphere (near the oil wells, a mature or a relict rhizosphere). This may indicate that the soil subjected to a long-term natural remediation may already have sufficient conditions for the growth and development some plants such as: field clover (*Trifolium arvense* L.), common starweed (*Stellaria media* L.), and fescue (*Festuca pratensis* L.) (Khan, 2005). The rhizosphere of these plants is usually rich in soil microorganisms including endophytes. The fungal and bacterial endophytes establish with these plants a close symbiosis (Prenafreda-Boldou et al., 2005; Partida-Martinez and Heil, 2011).

Endomycorrhiza dominates among green plants (at least in 70–80% of plants), and the degree of plant root colonization by mycorrhizal fungi and their activity undergo changes during the growing season (Khan, 2005). Arbuscular mycorrhizal fungi (AMF) are one of the most important biotic determinants of soil environmental quality (Jamiołkowska et al., 2018). They play a very important role in ecosystems and, through the production of glomalin related soil proteins (GRSP), can exert a positive effect on plant growth and health (Gałązka and Gawryjołek, 2015). Despite the extensive knowledge of the beneficial effects of AMF on plant growth and development, relatively little is known about the additional environmental function served by the fungi associated with the production of GRSP and its presence in the soil. GRSP are stable, water-insoluble, and degradation-resistant molecules stabilizing soil aggregates (Khan, 2005). Such properties make GRSP very stable compounds ideal to protect soil from degradation (Blaudez et al., 2000; Johnson et al., 2003). Fungi producing GRSP are also actively involved in the bioremediation process (Gonzalez-Chavez et al., 2004; Jamiołkowska et al., 2017; Pathak et al., 2017).

Investigations and selection of fungi with the highest bioremediation efficiency should therefore be related to the effectiveness of these strains in the competition with other fungi that may colonize the same area (Pathak et al., 2017). There are many examples of PAH-utilizing fungi in the literature. The best known examples are: *Glomus mosseare* and *Glomus etunicatum* increase fluorene and phenanthrene utilization in roots of alfaalfa (Gao et al., 2010), *Aspergillus*, *Paecilomyces*, *Penicillum*, *Cladohpialosphora*, *Exophila*, *Phialophora*, *Paecilomyces* in soil polluted with gasoline and petroleum (Winquist et al., 2014). Moreover, the bioremediation potential is known of *Sporothrix* and *Teberdinia* in toluene polluted soil (Prenafreda-Boldou et al., 2005). Thus, it is necessary to develop inexpensive and effective techniques for soil remediation and immobilization of toxic compounds at deposition sites (Johnsen and Karlson, 2007).

Evaluation of the microbiome and mycobiome of contaminated sites and determination of synergistic effects of microorganisms in an optimal plant-microsymbiont system may initiate work on the development of an effective and environmentally friendly co-metabolic system for reclamation of soils contaminated with petroleum-derived substances and trace elements. Biological activity restoration in long-term contaminated areas is very difficult and usually requires repetitive human intervention. The first report on the structural and functional characterization of the bacterial microbiome in samples of petroleum-contaminated soil was presented by Gałązka et al. (2018). The present study emphasizes assessment of the fungal role in long-term natural bioremediation of persistently contaminated soils.

The research hypothesis assumed that long-term natural bioremediation of crude oil contaminated soils can contribute to intensive development of a unique fungal community adapted to the contamination conditions. Due to the ruderal vegetation present in the contaminated soil, mycorrhiza may also develop in these soils. The aim of the study was to evaluate the fungal community and functional diversity in long-term crude-oil contaminated soils as the potential bioremediators of oil contaminated sites as well as the involvement of mycorrhiza fungi and the glomalin related soil protein content. Evaluation of the fungal function in long-term naturally remediated soil is the basis for further research to select strains that are active in bioremediation. Such fungi could then be used in bioremediation processes.

## Materials and Methods

### Soil Samples and Glomalin-Related Soil Proteins Analysis

Soil samples were collected according to the methodology described previously by Gałązka et al. (2018). Light loamy sand with long-term petroleum contamination was taken from oil wells in Węglówka (Podkarpackie Voivodship, Poland) (Gałązka et al., 2018). The Oil Mine in Węglówka was founded in 1881 and was active until 1950. Therefore, the area after the mine was heavily contaminated and degraded. From 1950, oil production was not carried out anymore and the site itself has naturally remedied itself to the present day. Nevertheless, from the oil mine closure to the present time, crude oil has flowed spontaneously from the oil wells. Constant crude oil flow causes permanent contamination of the area with simultaneously occurring natural remediation. Soil samples were collected from three oil wells: Oil well no1 (49°76′60"N, 21°79′69"E); Oil well no2 (49°76′90"N, 21°77′60"E), and Oil well no3 (49°76′80"N, 21°77′45"E). Soils were collected from the 0–20 cm layer in three biological replicates and at two distances (Table 1): three biological replications of pooled samples within a 0.5 m radius of the oil wells (Oil Well Petroleum – OWP) and three biological replications of pooled samples within a 3 m radius from the oil wells (Oil Well – OW). The precise methodology of sampling is given in the paper Gałązka et al. (2018). The soil samples were sieved through a 2 mm sieve. The samples were stored in a refrigerator (4°C) until analysis. The area from which soil samples were collected at a distance of 3 m from the oil well was overgrown with meadow vegetation, while the soil taken directly under the oil well was characterized by little or no vegetation. The vegetation present at the sampling site was not characterized. However, within two meters from the oil well, the soil was not overgrown with vegetation and strongly degraded.

**TABLE 1:** The location of the soil samples.

Sample	Distance (Radius)	Environment	SRA NCBI ID
OWP1_ITS	0.5 m from oil well no 1, Weglowka, Poland	Soil under oil well	SRX4157197
OWP2_ITS	0.5 m from oil well no 2, Weglowka, Poland	Soil under oil well	SRX4157195
OWP3_ITS	0.5 m from oil well no 3, Weglowka, Poland	Soil under oil well	SRX4157193
OW1_ITS	3 m from oil well no 1, Weglowka, Poland	Grassland soil	SRX4157196
OW2_ITS	3 m from oil well no 2, Weglowka, Poland	Grassland soil	SRX4157194
OW3_ITS	3 m from oil well no 3, Weglowka, Poland	Grassland soil	SRX4157192

*Summary of deposited sequencing data. Public data was deposited in the NCBI Sequence Read Archive (SRA) under accession number SRP14956 (https://www.ncbi.nlm.nih.gov/sra/SRP149656).*

GRSP content was determined as proposed by Wright et al. (1996) with modifications described by Gałązka et al. (2017). The glomalin-related soil proteins concentrations were determined spectrophotometrically as both total glomalin-related soil proteins (T-GRSP) and easily extractable glomalin-related soil proteins (EE-GRSP). TG-GRSP was obtained by repeated extraction from 1 g of ground dry-sieved soil with 8 mL of 50 mM citrate, pH 8.0 at 121°C for 60 min. The EE-GRSP protein was extracted from 1 g of ground dry-sieved soil with 8 mL of 20 mM citrate, pH 7.0 at 121°C for 30 min. After each autoclaving cycle, the supernatant was removed by centrifugation at 4000 rpm for 15 min and stored. Extracts from each cycle were pooled, centrifuged at 9000 rpm for 5 min to remove soil particles and then analyzed. Extracts from each replicate were pooled and analyzed, and the protein in the supernatant was determined by the Bradford dye-binding assay with bovine serum albumin as the standard on a 96-plate reader (Victor, Perkin Elmer).

### Determination of PAHs

Before extraction, all samples were dried at 25 ± 1°C and sieved through a 2 mm sieve. Subsamples intended for analyses of 16 PAHs were ground to obtain a 0.1 mm particle size fraction and subjected to the following procedure. A 20-g sample was fortified with a known amount of internal standard that contained a mixture of d8-naphtalene, d10-acenaphtene, d10-phenanthrene, d12-chrysene and d12-perylene and extracted in an Accelerated Solvent Extractor (ASE200, Dionex) with dichloromethane. The next step included clean-up on deactivated silica gel with a conventional method using solvents with different polarity, followed by a decreasing volume of an eluent on a rotary evaporator to obtain a sample dissolved in hexane. PAH determination was completed using a gas chromatograph (Agilent 6890) equipped with a mass spectrometer (Agilent 5973 Network) and an autosampler (Agilent 7683 Series B). A detailed description of PAH resolution and quality control conditions are described by Maliszewska-Kordybach et al. (2009). PAH determination included 15 compounds from the US-EPA list (1997) except naphthalene, and groups of 3-, 4- and 5 + 6-ringed compounds were distinguished. The 3-ringed compounds included acenaphtene, acenaphtylene, fluorene, phenanthrene and anthracene. The 4-ringed compounds were fluoranthene, pyrene, chrysene and benzo[a]anthracene; the 5 + 6-ringed compounds were represented by benzo[b]fluoranthene, benzo[k]fluoranthene, benzo[a]pyrene, dibenzo[a,h]anthracene, indeno[1,2,3-cd]pyrene and benzo[g,h,i]perylene. Low molecular (LM) PAHs included the 3- and 4-ringed compounds, while the 5 + 6-ringed compounds constituted high molecular (HM) PAHs. The results of extraction of petroleum hydrocarbons were presented in Gałązka et al. (2018).

### Determination of the Trace Elements Content – Inductively Coupled Plasma Mass Spectrometry (ICP-MS)

Soil samples were digested in aqua regia using a Mars Xpress microwave digestion system by CEM Inc. equipped with middle pressure (32 bar) vessels. Air-dried soil was used; the sample weight was 0.5 g. Ten mL aqua regia, prepared from instra-analyzed grade nitric and hydrochloric acids from J.T. Baker, was added to every digestion vessel. After digestion, samples were added to 50 mL falcon vials and diluted to 50 mL with MilliQ water.

Immediately before analysis, samples were further diluted 1:10 in MilliQ grade water. To ensure the quality of the analysis, the same procedure was performed for blank and certified reference materials. Analysis was performed on an Agilent 7500ce ICP-MS in the presence of 45Sc, 89Y and 159Tb as internal standards to minimize the matrix effect and ensure long-term stability. The accuracy was 10%, and the quantification limits were set at 0.01 mg ⋅ kg^–1^.

### Functional Diversity Analysis Using Biolog FFPlates

The Biolog FFPlate method (Biolog Inc., Hayward, United States) was used to evaluate the metabolic potential of the fungal communities in the contaminated soils. The functional diversity analysis of fungi were determined using the Biolog FFPlate with 96 different carbon sources (Borowik et al., 2017, 2019). The 1 g of soil was transferred to flasks (each with 99 cm^3^ sterile 0.9% NaCl) and vortexed for 30 min at 150 rpm and 25°C. Nextthe samples were cooled for 30 min to 4°C and after that 100 mm^3^ was transferred to each well in a FFPlate and incubated in the dark at 25°C for 120 h. The experiment included three replications. The results were read on a MicroStation ID systems by Biolog^®^ an (λ = 490 nm; Gałązka et al., 2017). The most intensive carbon substrate metabolism was observed after 96–120 h incubation. The activities of fungi are based on all carbon sources and grouped sources defined as amines and amides, amino acids, carbohydrates, carboxylic acids and polymers (Preston-Mafham et al., 2002). Three FFPlates were used for each soil sample to obtain three replications as a result of analysis.

### Isolation of DNA and ITS Sequencing

DNA isolation from soil was performed according to the manufacturer’s instructions. The 350 mg of fresh soil was transferred into 1.5 mL tubes for extracting DNA with the FastDNA^TM^ SPIN Kit for Soil (MP Biomedical), DNA concentration were measured with a NanoDrop 1000 Spectrophotometer (Thermo Fisher Scientific). DNA was diluted with sterile MilliQ water to 10 ng μl^–1^ and sequenced at Genomed S.A (Warsaw, Poland) with 2 × 250 base pair (bp) paired-end technology using the Illumina MiSeq system. Amplification of the hypervariable ITS1 region was performed with Q5 Hot Start High-Fidelity 2x Master Mix, according to the manufacturer’s instructions, with ITS1Fl2 (5′-GAACCWGCGGARGGATCA-3′) and 5.8S (5v-CGCTGCGTTCTTCATCG-3′) primers (Schmidt et al., 2013). PCR reaction using Q5 Hotstart High-Fidelity 2x Master Mix (New England Biolabs), 5.8S and ITS1FI2 primers extended with an adapter sequence compatible with Nexter XT indexes, according to the following conditions: input ∼15 ng DNA, initial denaturation (98°C, 30 s), denaturation (98°C, 10 s), primer attachment 52°C, 30 s), extension (72°C, 20 s), final extension (72°C, 2 min). PCR products were purified with AMPure Beads XP (Beckman Coulter) and indexed with the Nextera XT Index Kit (Illumina) in a 7-cycle amplification reaction. Raw fastq files (both forward and reverse) were deposited in the NCBI SRA database (Table 1).

### Bioinformatics and Statistical Analyses

All steps mentioned below were executed in R version 3.4.3 (R Core Team, 2016). Exact amplicon sequence variants (ASVs) were resolved from forward reads using the DADA2 version 1.6 package (Callahan et al., 2016). Sequences were trimmed to 250 nucleotides (nt); the first-left 20 nt were removed (these contained primers and low quality bases). Filtering the sequences was set to: *maxN* = 0, *maxEE* = 5, and *truncQ* = 2. The sequences were dereplicated using *derepFastq* with default parameters, and exact sequence variants were resolved using *dada*. Next, *removeBimeraDenovo* was used to remove chimeric sequences by applying the consensus method. After quality filtering and chimeric sequence removal from forward reads, an average of 85% (± 3%) sequences remained for further analysis. For comparison, with paired-end reads, only 55% (± 7%) remained and were successfully merged; thus, only forward reads were subsequently chosen for analysis. Taxonomy was assigned against the latest version of the UNITE database (version 7.2, built 2017.12.01) with the dynamic clustering thresholds, using a Naïve Bayesian Classifier (Wang et al., 2007). The resulting taxonomy was converted into the phyloseq object and imported in the phyloseq package (McMurdie and Holmes, 2013). All taxa other than Fungi were removed.

The main statistical analyses were performed using STATISTICA 10 (Stat. Soft. Inc., United States). Collected data was subjected to analysis of variance (ANOVA) for comparison of means. Significant differences were calculated according to Tukey’s *post hoc* test at a *P* < 0.05 significance level. The Pearson’s correlation coefficient (*P* ≤ 0.05) of abundance of fungi and PAHs and trace elements contents were tested. The results of fungal amplicon libraries were also submitted to principal component analysis (PCA) to determine the correlations between the fungal core metagenome and soils collected from different oil wells. PAHs degrading candidates were determined on the basis of the selection from the NCBI database of the sequences of fungi which were confirmed as the PAHs bioremediators. On this basis, the percentage abundance of these genera in the control and contaminated samples was compared.

## Results

### Chemical Analysis of Soil Samples

The shape of PAH group contributions to total content was similar for all samples and independent of the distance from the oil well (Table 2). The samples taken directly from the oil well showed a higher concentration of PAHs. In all samples, the most abundant PAHs were 5 + 6 hydrocarbons (781–1480 μg ⋅ kg^–1^), while the 3-ringed group represented the smallest amount (296–721 μg ⋅ kg^–1^). The share of 3- and 4-ringed PAHs was higher as the distance from the oil well increased (except for location 1, where the 3-ringed PAH contribution was 1.5-times higher close to the oil well). This finding indicates more effective degradation processes occur closer to the oil wells.

**TABLE 2:** Evaluation of PAH group loads in soils (*n* = 3).

PAH groups	Soil samples					
	OWP1	OWP2	OWP3	OW1	OW2	OW3
**Content (μg kg**^–^**^1^)**						
Σ16PAHs	2849^a^ ± 570	2938^a^ ± 588	2749^b^ ± 550	1464^d^ ± 293	1794^c^ ± 359	2066^b^ ± 413
3-ringed	382^c^ ± 46	483^b^ ± 58	721^a^ ± 87	310^d^ ± 37	296^d^ ± 36	575^b^ ± 69
4-ringed	995^a^ ± 289	975^a^ ± 283	641^b^ ± 186	372^c^ ± 108	620^b^ ± 180	577^b^ ± 167
5 + 6-ringed	1472^a^ ± 339	1480^a^ ± 340	1387^a^ ± 319	781^b^ ± 180	877^b^ ± 202	914^b^ ± 210
**Contribution to total (%)**						
3-ringed	13^b^ ± 0.7	16.4^b^ ± 1.3	26^a^ ± 1.3	21^a^ ± 3.5	17^b^ ± 2.0	28^a^ ± 1.4
4-ringed	35^a^ ± 0.6	33^a^ ± 1.0	23^b^ ± 1.6	25^c^ ± 3.1	35^a^ ± 1.0	28^b^ ± 1.5
5 + 6-ringed	52^a^ ± 0.8	50^a^ ± 0.6	50^a^ ± 1.3	53^a^ ± 2.3	49^a^ ± 3.4	44^b^ ± 0.9

*Treatment means separated by different letters (a,b,c,d) are significantly different (Tukey’s mean separation test, *p* < 0.05).*

Differences in the content of selected trace elements are also presented (Table 3). Our previous study (Gałązka et al., 2018) provided the content of basic trace elements and microelements in long-term crude-oil contaminated soil. The current research focused on lessknown, unusual trace elements such as: beryllium, vanadium, arsenic, selenium, strontium, antimony, barium, lanthanum, cerium, europium, gadolinium, thallium, and bismuth. The soil samples collected directly from the oil well (especially OWP1 and OWP3) had a lower concentration of most of the tested trace elements such as: beryllium, vanadium, arsenic, barium, lanthanum, and cerium (Table 3). Generally a lower concentration of trace elements in soils situated nearer to the oil well (OWP) were found than those situated further away (OW). It may be due to the fact that these trace elements are washed away with water from the contamination site. At a distance of 3 meters from the oil wells, the area is overgrown with plants, so the trace elements can be accumulated in the plant. For example the highest concentration of strontium was presented in sample OWP2 (140.29 mg ⋅ kg^–1^). Similarly, the strontium concentration in the OWP1 sample was 30.73 mg ⋅ kg^–1^, while in the control sample (OW1) the concentration was twice as high. For example, it was found that the beryllium content in the OWP1 sample was 0.21 mg ⋅ kg^–1^, while the beryllium content in the OW1 sample increased to 1.54 mg ⋅ kg^–1^. Similar results were obtained for such trace elements as arsenic and barium (Table 3). The content of the trace elements was distributed very unevenly in the samples. This may indicate different availability of elements in the soil as well as their leaching to deeper soil layers.

**TABLE 3:** Evaluation of trace element loads in soils (*n* = 3).

	OWP1	OWP2	OWP3	OW1	OW2	OW3
**Trace elements content [mg kg**^–^**^1^]**						
Be (beryllium)	0.27^c^	1.74^a^	0.41^c^	1.54^a^	0.85^b^	1.23^ab^
V (vanadium)	12.65^c^	28.28^b^	14.45^c^	28.96^b^	43.47^a^	48.96^a^
As (arsenic)	2.81^c^	8.53^a^	2.67^c^	5.63^b^	7.32^a^	7.60^a^
Se (selenium)	0.12^b^	0.36^a^	0.14^b^	0.44^a^	0.36*a*	0.43^a^
Sr (strontium)	30.73^c^	140.29^a^	32.86^c^	74.67^b^	47.19^c^	118.53^a^
Ag (silver)	0.05^b^	0.08^a^	0.03^b^	0.16^a^	0.08^a^	0.10^a^
Sb (antimony)	0.18^d^	1.04^b^	0.45^c^	1.29^a^	1.77^a^	1.18^a^
Ba (barium)	39.69^d^	170.47^b^	86.27^c^	172.03^b^	116.77^c^	312.51^a^
La (lanthanum)	6.81^c^	11.47^b^	7.20^c^	15.63^b^	24.04^a^	20.99^a^
Ce (cerium)	13.91^c^	23.16^c^	14.80^c^	32.65^b^	52.22^a^	45.19^a^
Eu (europium)	0.22^b^	0.46^b^	0.22^b^	0.55^a^	0.75^a^	0.77^a^
Gd (gadolinium)	1.17^c^	2.21^b^	1.15^c^	2.70^b^	4.24^a^	3.74^a^
Tl (thallium)	0.06^b^	0.29^a^	0.09^b^	0.26^a^	0.29^a^	0.35^a^
Bi (bismuth)	0.03^b^	0.15^a^	0.06^b^	0.15^a^	0.19^a^	0.19^a^

*For each trace element, different superscript letters indicate a statistically significant difference in the content.*

### Functional Diversity of Soils

**FIGURE 1:**
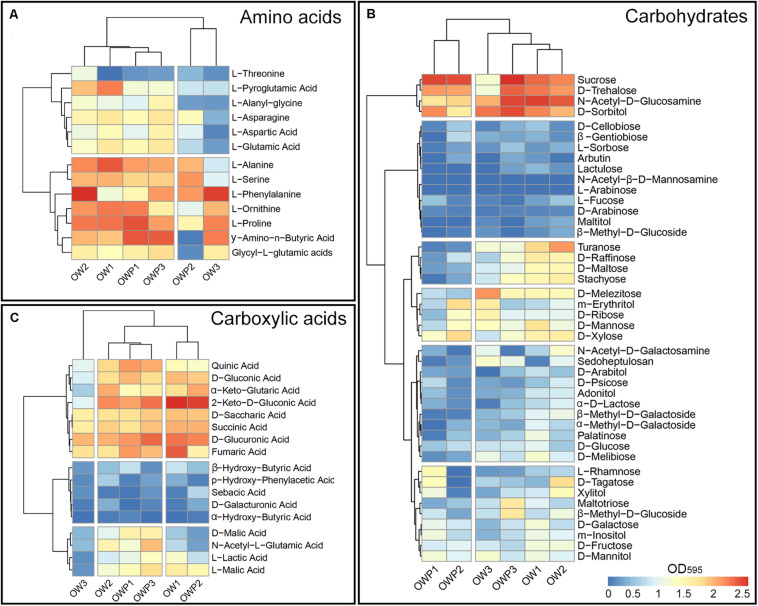
Heatmaps for the carbon utilization patterns of the substrates from the Biolog FFPlates data incubated for 120 h from soil samples: **(A)** Amino acids. **(B)** Carbohydrates. **(C)** Carboxylic acids (*n* = 3). Legend: high substrate utilization activities (red), low substrate utilization activities (blue).

From the 95 different carbon sources, the highest rates of substrate utilization were recorded for the soil collected 3 m from the oil wells (Figure 1). Further, amino acids (Figure 1A), carbohydrates (Figure 1B) and carboxylic acids (Figure 1C) had the highest metabolic activity in samples collected 3m from the oil wells. Samples OW1 and OW2 had the highest activity from the use of amino acids as the carbon source (Figure 1A). The most frequently utilized amino acids were L-Alanine, L-Serine, L-Phenylalanine, L-Ornithine, L-Proline, and Glycyl-L-glutamic acids. These amino acids were also most commonly utilized by fungi in samples taken from under the crude oil wells (OWP1, OWP2, and OWP3). The L-Threonine was the least used. There was a similar relationship in the case of carbohydrates as a carbon source. Samples OW1 and OW2 had the highest activity from the use of carbohydrates as the carbon source (Figure 1B). The highest utilization of carbohydrates was observed also in sample OWP1 in such sources as: D-Trehalose, N-Acetyl-D-Glucosamine and D-sorbitol. To the least utilized carbohydrates included: D-Cellobiose, β –Gentiobiose, L-Sorbose, Arbutin, Lactulose, N-Acetyl-β-D-Mannosamine, L-Arabinose, L-Fucose, D-Arabinose, Malitol, and β-Methyl-D-Glucoside. From the most frequently utilized carboxylic acids were: Quinic Acid, D-Gluconic Acid, α-Keto-Glutanic Acid, 2-Keto-D-Gluconic Acid, D-Saccharic Acid, Succinic Acid, D-Glucuronic Acid, and Fumaric Acid. The OW3 sample was characterized by lowest utilization of carboxylic acids (Figure 1C). Biodiversity indicators were determined on the basis of the results of assessment of metabolic biodiversity and sequencing (Table 4). The soil samples were characterized by variable biodiversity indicators. Nevertheless, samples taken directly from the oil wells exhibited greater biodiversity. Indeed, the highest Shannon indexes obtained by Biolog FFPlate method were observed in OWP1 (*H*’ = 4.45), OWP2 (*H*’ = 4.52) and OWP3 (*H*’ = 4.61), while in the control samples this index was much lower (Table 4). Similar results were obtained for substrate richness (R) and substrate evenness (E). In the case of average well-color development (AWCD_590_) obtained in the Biolog FFPlates incubated for 120 h the results were the opposite. The control samples showed the highest AWCD index. This means that in the control samples there were characterized by higher fungal activity but much less diversity. Similar results were obtained for the determination of biodiversity indicators based on data from the Next Generation Sequencing. The highest Shannon indexes obtained by ITS NGS method were observed in OWP1 (*H’* = 4.86), OWP2 (*H’* = 4.91), OWP3 (*H*’ = 4.65), and OW2 (*H’* = 4.84), while in the samples OW1 and OW3 this index was insignificantly lower (4.52 and 4.41). Completely different results were obtained for the Fisher index, where the highest values of this index were obtained for the OWP1, OW1, and OW3 samples. However, in the case of the Simpson index, the highest value of the index was obtained for the OWP2 and OW2 samples.

**TABLE 4:** Changes in fungal metabolic diversity in soil contaminated with crude oil after long-term natural bioremediation as evaluated by: Shannon’s general diversity index (*H*’), substrate richness (R), substrate evenness (E), and average well-color development (AWCD_590_) obtained in the Biolog FFPlates incubated for 120 h and ITS next generation sequencing (total richness indicators calculated on the basis of all sequences = real diversity).

Sample	*H’*	R	*E*	AWCD_590____
	
	Biolog FFPlate			
OWP1	4.45^a^	95^a^	0.994^a^	0.775^b^
OWP2	4.52^a^	94^a^	0.982^a^	0.681^b^
OWP3	4.61^a^	94^a^	0.986	0.895^ab^
OW1	4.01^b^	92^ab^	0.951^b^	0.972^a^
OW2	4.12^b^	90^b^	0.942^b^	0.998^a^
OW3	4.21^b^	91^b^	0.956^b^	0.979^a^

	**ITS NGS**			
	***H’***	**Chao1**	**Simpson**	**Fisher**

OWP1	4.86^a^	512^b^	49.53^b^	88.70^a^
OWP2	4.91^a^	445^b^	64.93^a^	76.71^b^
OWP3	4.65^ab^	447^b^	44.31^b^	68.87^b^
OW1	4.52^b^	604^a^	27.48^c^	92.56^a^
OW2	4.84^a^	318^c^	63.83^a^	46.43^c^
OW3	4.41^b^	585^a^	27.95^c^	89.79^a^

*Treatment means separated by different letters (a,b,c,d) are significantly different (Tukey’s mean separation test, *p* < 0.05).*

### Glomalin Related Soil Protein Content

There was a statistically significant increase in T-GRSP content in soil samples taken 3 m from the crude oil wells (Table 5). The EE-GRSP content was also significantly higher in soil samples taken 3 m from the wells. Consistently, the T-GRSP and EE-GRSP contents were lower in soil samples taken directly from the crude oil well. The highest T-GRSP content was observed in OW3 and lowest in OWP1. The highest EE-GRSP content was observed in OW2 and the lowest in OWP1.

**TABLE 5:** The total and easily-extractable glomalin-related soil proteins (TG-GRSP and EEG-GRSP, respectively) content (*n* = 3).

Sample	T-GRSP	EE-GRSP
OWP1	3,306^c^ ± 0.21	1,051^c^ ± 0.09
OWP2	4,759^b^ ± 0.05	2,219^b^ ± 0.12
OWP3	5,785^ab^ ± 0.12	2,488^b^ ± 0.04
OW1	5,116^b^ ± 0.03	2,375^ab^ ± 0.16
OW2	8,245^a^ ± 0.12	3,640^a^ ± 0.17
OW3	6,463^ab^ ± 0.08	2,916^ab^ ± 0.08

*^*a*^Total glomalin-related soil proteins (TG-GRSP). ^*b*^Easily extractable glomalin-related soil proteins (EEG-GRSP). ^*c*^Treatment means separated by different letters (a,b,c,d) are significantly different (Tukey’s mean separation test, *p* < 0.05)*

This data may indicate that the control soil (OW) subjected to a long-term natural remediation may already have sufficient conditions for the growth and development of plants and mycorrhizal fungi to allow them to develop sufficiently while using PAH as the sole carbon and energy source.

### Fungal Genetic Diversity

**FIGURE 2:**
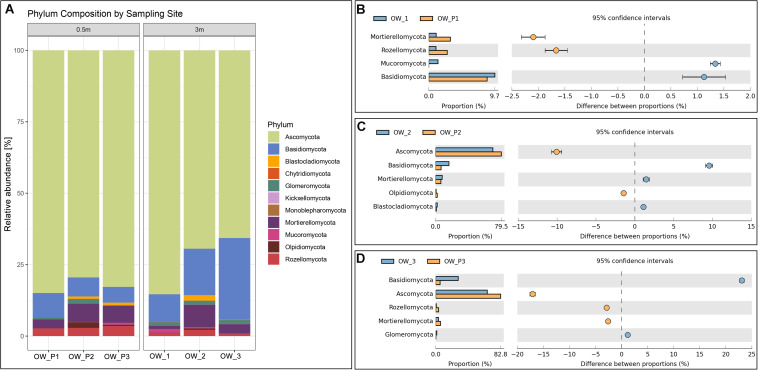
Relative abundance of dominant fungal phyla in the different soil samples (percentage of sequences) based on next generation sequencing. **(A)** Phylum composition by sampling site. **(B)** Comparison of dominant phyla between OW1 and OWP1. **(C)** Comparison of dominant phyla between OW2 and OWP2. **(D)** Comparison of dominant phyla between OW3 and OWP3.

**FIGURE 3:**
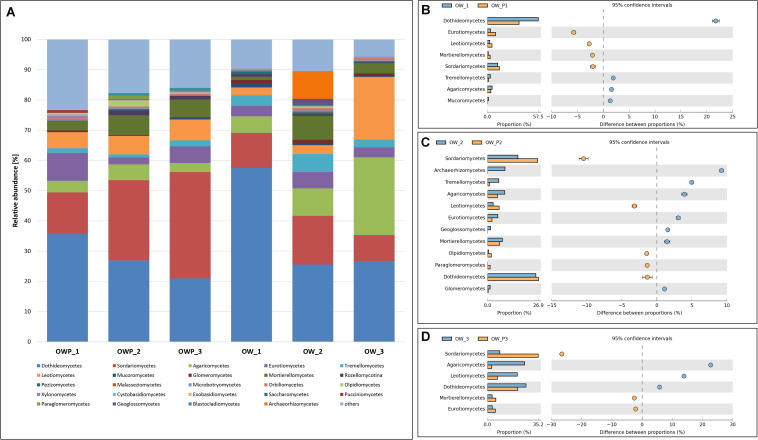
Relative abundance of dominant fungal classes in the different soil samples (percentage of sequences) based on next generation sequencing. Classifications with less than 1% abundance are gathered into the category “other.” **(A)** Class composition by sampling site. **(B)** Comparison of dominant classes between OW1 and OWP1. **(C)** Comparison of dominant classes between OW2 and OWP2. **(D)** Comparison of dominant classes between OW3 and OWP3.

The two main phyla observed in the analyzed samples were *Ascomycota* and *Basidiomycota* (Figure 3A). The relative abundance of *Ascomycota* was higher in the group of samples collected directly from the oil wells. In addition to the two main phyla, *Blastocladiomycota*, *Chyridiomycota*, *Kickellomycota*, *Monoblepharomycota*, and *Mycoromycota* were also observed (Figure 2A). *Ascomycota* was present at the same level of abundance in OWP1 and WP1 samples (Figure 3A) and was in significantly higher abundance compared to OWP2, OWP3 and OW2 and OW3. The Figures 3C,D showed the dominant taxa of *Ascomycota* is samples collected 3 m from oil wells (OWP2 and OWP3). *Ascomycota* was higher in OWP2 compared to OW2. The same was confirmed for the samples OWP3 and OW3. The higher relative abundance of *Basidiomycota* was observed in sample OW2 and OW3 (Figure 2A). By comparing the samples with pairs of distances (OWP1 – OW1), it was found that OWP1 sample was characterized higher abundance of *Mortierellomucota* and *Rozellomycota* compered to OW1 sample (Figure 2B). OWP2 and OWP3 samples were characterized higher abundance of *Ascomycota* (Figures 2B,C,D) but lower abundance of *Basidiomycota*.

For the class taxonomic level, samples taken directly from oil well had a more unified composition compared to samples taken 3 m from the oil wells (Figure 3A). In OW1, OW2, and OW3, where remediation had occurred for many years, there was already a natural remediation, denoted by marked variety and variability of the sequenced classes. The dominant taxa were: *Dothideomycetes*, *Sordariomycetes*, *Agaricomycetes*, *Eurotiomycetes*, *Tremellomycetes*, *Leotiomycetes*, *Mucoromycetes*, and *Glomeromycetes*. The fungi from *Glomeromycetes* are responsible for GRSP production. These fungi occurred in greater numbers in sample, where the soil surface was covered with a variety of grass vegetation (Figures 3B,C,D). Percent of relative abundance of *Glomeromycetes* in sample was equal 1.14, 1.38, 0.95, 0.59, 0.29, and 0.07%, respectively for OW1, OW2, OW3, OWP1, OWP2, and OWP3.

**FIGURE 4:**
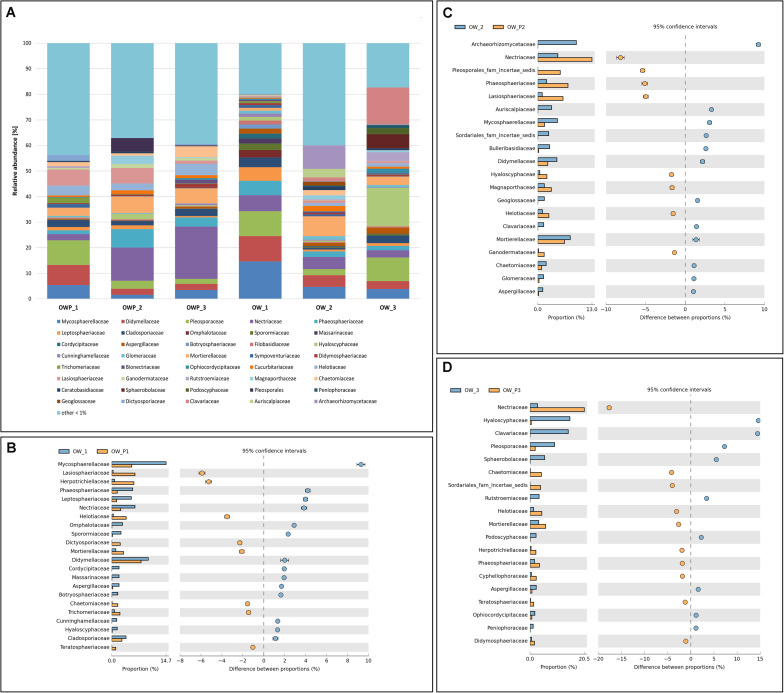
Relative abundance of dominant fungal families in the different soil samples (percentage of sequences) based on next generation sequencing. Classifications with less than 1% abundance are gathered into the category “other.” **(A)** Family composition by sampling site. **(B)** Comparison of dominant families between OW1 and OWP1. **(C)** Comparison of dominant families between OW2 and OWP2. **(D)** Comparison of dominant family between OW3 and OWP3.

**FIGURE 5:**
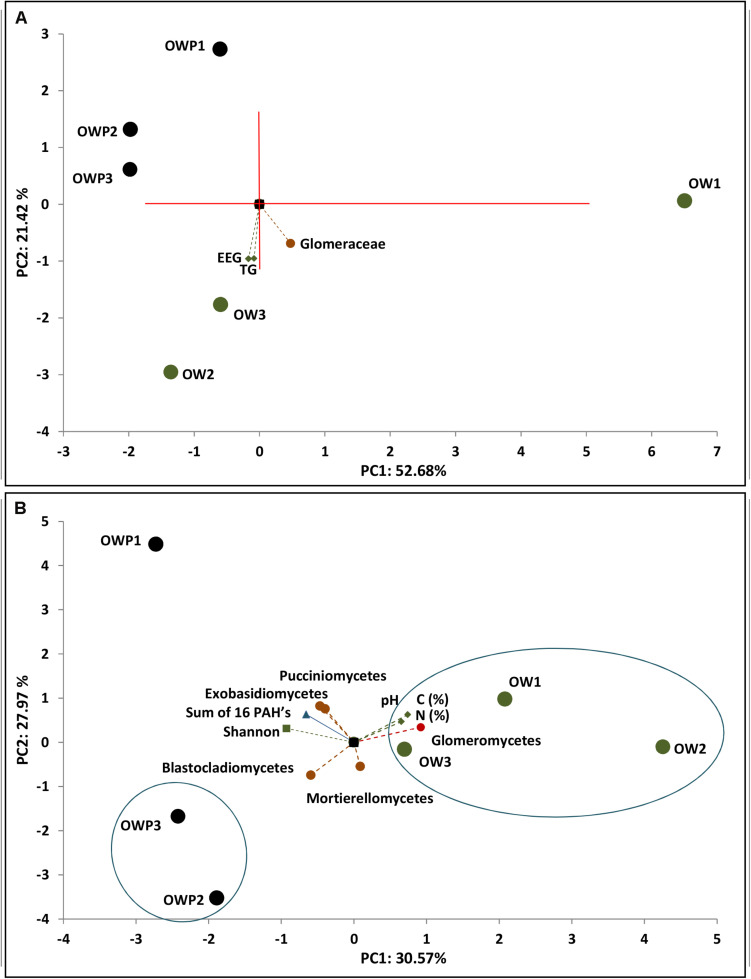
Principal component analysis of glomalin-related soil proteins content, sum of 16 PAHs and soil samples (*n* = 3). **(A)** Correlation of TG-GRSP content and family *Glomeraceae*. **(B)** Correlation of glomalin-related soil proteins content and main genera and biodiversity indices.

At the family level, samples OW1, OW2, and OW3 were characterized by greater diversity (Figure 4A). OW1 was characterized by great family richness and diversity (Figure 4B). OWP1, OWP2, and OWP3 were also characterized by high variability, with the majority of fungi from the *Pleosporacea*e family (Figures 4B–D). In the samples taken directly from the crude oil wells, there were families whose representatives actively use PAHs as the sole carbon and energy source. In OW1, the predominant taxawere *Mycosparellaceae* and *Didymellaceae* (Figure 5B). In OW2 and OWP2, the families *Archaeorhizomycetaceae*, *Netriaceae*, and *Mortierellaceae* dominated (Figure 5). In OW3 and OWP3, the dominant families were *Nertiaceae*, *Hyaloscyphaceae*, and *Pleosporaceae* (Figure 4D).

The PCA analyses indicated strong correlations between the T-GRSP and EE-GRSP with the *Glomeraceae* and *Glomeromycetes* families, i.e., glomalin-producing mycorrhizal fungi (Figure 5A). Glomalin contents were strongly correlated with samples taken 3 m from the crude oil well. Strong correlation in the fungal community composition of samples taken directly from the crude oil well between the sum of 16 PAHs, total carbon and total nitrogen content, pH and the Shannon biodiversity index and with *Mortierellomycetes*, *Exobasidiomycetes*, *Pucciniomycetes*, and *Blastocladiomycetes* fungi (Figure 5B).

**FIGURE 6:**
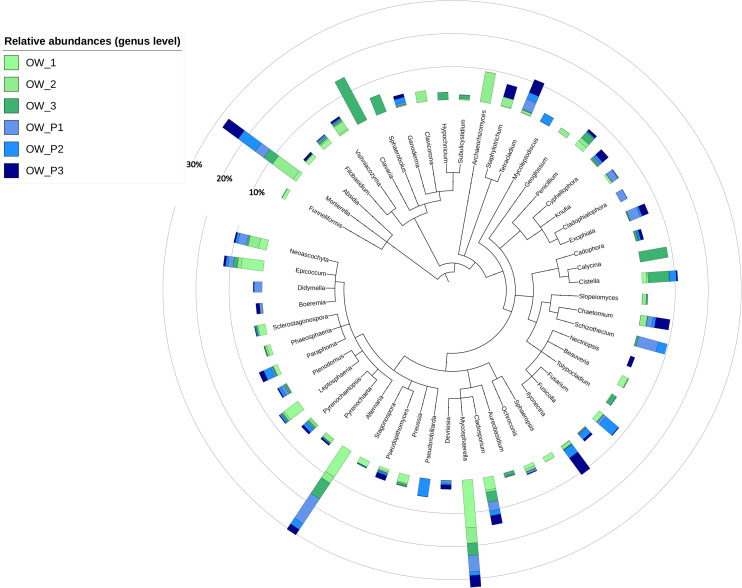
Top genera composition in soil samples.

At the genus level, the extent of diversity depended on the sampling distance (Figure 6). In OW1 taken 3 m from the oil well, the genus *Mycospaerella* dominated, and in the sample taken directly from the extract (OWP1) the following genera predominated: *Schizothecium*, *Exophiala*, *Tetracladium*, *Didymella*, and *Cladophialophora* (Figure 6). In turn, OW2 was dominated by *Archaeorhizomyces*, and *Pseudorobillardia* and *Giberella* dominated in OWP2 (Figure 6). The most abundant genera in the OW3 belonged to *Clavia*, while in OWP3 *Fusarium*, *Chaetomium*, *Tetracaldium*, and *Staphylotrichum* genera were found. Analysing relative abundances at the genus level for all samples, the most abundant genera were *Mortierella*, *Clavaria*, *Mycosphaerella*, *Alternaria*, *Cladosporium*, *Epicoccum*, and *Neoasco*. Within the samples, there was high variability that depended on the sampling distance and the crude oil well. The genus of *Tetracladium*, *Exophilia*, *Schizothecium*, and *Ilyonectria* identified directly from the crude oil wells could be the most interesting in terms of their application in bioremediation (Figure 6).

No statistically significant correlations were obtained in most of the trace elements and selected fungi genera (Table 6). The examples of selected correlations between the selected genera and the trace elements content was presented in Table 6. All correlations are presented in Supplementary Material – Table 1. The strong positive correlations were confirmed between: *Sclerostagonospora* and Pb (*r* = 0.825), *Saccharicola* and Pb (*r* = 0.811), *Penicillium* and K (*r* = 0.854), *Mortierella* and La (*r* = 0.858), *Phaeosphaeria* and Mn (*r* = 0.837) and Sn (*r* = 0.897), *Cistella* and Be (*r* = 0.927) (Table 6).

**TABLE 6:** Pearson’s correlation coefficient (*P* ≤ 0.05) of selected fungi genera and trace elements (data marked in bold are statistically significant).

	*Mycosphaerella*	*Epicoccum*	*Leptosphaeria*	*Preussia*	*Sclerostagonospora*	*Beauveria*	*Saccharicola*	*Sphaeropsis*	*Penicillium*	*Filobasidium*	*Cistella*	*Absidia*	*Mortierella*	*Phaeosphaeria*
Li	0.009	0.055	0.091	0.208	0.153	0.082	0.063	0.043	0.786	0.191	0.778	–0.084	0.026	0.367
Cr	–0.275	–0.210	–0.142	–0.062	–0.062	–0.149	–0.147	–0.193	0.584	–0.091	0.731	–0.284	0.300	0.132
Mn	0.545	0.603	0.670	0.678	0.722	0.677	0.641	0.622	0.571	0.669	0.289	0.388	–0.165	**0.837**
Co	–0.104	–0.054	0.012	0.083	0.087	0.014	–0.014	–0.045	0.613	0.070	0.626	–0.196	0.255	0.322
Ni	–0.253	–0.147	–0.075	–0.025	0.005	–0.088	–0.059	–0.130	0.467	–0.029	0.703	–0.305	0.250	0.133
Cu	–0.174	–0.009	0.092	0.089	0.179	0.067	0.161	0.049	0.316	0.050	0.651	–0.084	0.121	0.117
Zn	–0.520	–0.455	–0.338	–0.460	–0.279	–0.337	–0.249	–0.325	–0.476	–0.576	–0.165	–0.071	0.541	–0.476
Mo	–0.419	–0.235	–0.126	–0.231	–0.053	–0.146	–0.021	–0.159	–0.288	–0.237	0.233	–0.302	0.345	–0.232
Cd	0.007	–0.100	0.008	–0.005	0.072	0.055	–0.053	0.000	0.278	–0.157	–0.163	0.291	0.549	0.299
Sn	0.588	0.583	0.657	0.661	0.707	0.682	0.597	0.616	0.573	0.622	0.101	0.480	–0.044	**0.897**
Pb	0.630	0.689	0.790	0.657	**0.825**	0.805	**0.811**	0.780	–0.047	0.596	–0.352	0.712	–0.142	0.686
Na	0.168	0.365	0.442	0.392	0.499	0.409	0.531	0.410	0.139	0.390	0.432	0.171	–0.226	0.290
Mg	0.334	0.481	0.540	0.580	0.598	0.505	0.599	0.518	0.583	0.523	0.689	0.413	–0.414	0.462
Al	–0.538	–0.501	–0.479	–0.600	–0.489	–0.486	–0.392	–0.444	**−0.818**	–0.624	–0.417	–0.257	0.316	–0.754
K	0.566	0.454	0.417	0.561	0.413	0.434	0.307	0.406	**0.854**	0.534	0.287	0.413	–0.313	0.704
Ca	–0.264	–0.428	–0.451	–0.480	–0.486	–0.415	–0.496	–0.409	–0.420	–0.529	–0.629	–0.075	0.391	–0.408
Fe	–0.214	–0.316	–0.375	–0.397	–0.433	–0.368	–0.370	–0.316	–0.496	–0.427	–0.496	–0.008	0.038	–0.547
Be	–0.025	0.126	0.141	0.238	0.187	0.092	0.204	0.118	0.575	0.224	**0.927**	–0.006	–0.343	0.090
V	–0.053	–0.089	–0.227	–0.226	–0.325	–0.237	–0.236	–0.203	–0.496	–0.071	–0.350	–0.361	–0.279	–0.387
As	–0.029	–0.181	–0.182	–0.183	–0.188	–0.127	–0.303	–0.209	0.003	–0.140	–0.419	–0.255	0.512	0.199
Se	–0.052	–0.089	–0.227	–0.226	–0.325	–0.237	–0.236	–0.203	–0.496	–0.071	–0.350	–0.361	–0.279	–0.387
Sr	–0.741	–0.716	–0.694	–0.757	–0.688	–0.705	–0.613	–0.661	–0.615	–0.806	–0.149	–0.399	0.423	**−0.881**
Ag	**−0.958**	**−0.988**	**−0.997**	**−0.975**	**−0.987**	**−0.993**	**−0.990**	**−1.000**	–0.331	**−0.951**	0.087	**−0.885**	0.690	**−0.865**
Sb	0.233	0.371	0.442	0.466	0.509	0.422	0.471	0.394	0.537	0.457	0.621	0.156	–0.162	0.511
Ba	**−0.957**	**−0.988**	**−0.997**	**−0.975**	**−0.987**	**−0.994**	**−0.990**	**−1.000**	–0.334	**−0.952**	0.083	**−0.884**	0.690	**−0.867**
La	–0.662	–0.668	–0.588	–0.740	–0.559	–0.557	–0.561	–0.594	–0.787	–0.747	–0.567	–0.511	**0.858**	–0.557
Ce	–0.741	–0.716	–0.694	–0.757	–0.688	–0.705	–0.613	–0.661	–0.615	–0.806	–0.149	–0.399	0.423	**−0.881**
Eu	–0.830	–0.751	–0.742	–0.808	–0.739	–0.764	–0.647	–0.728	–0.678	–0.788	–0.025	–0.652	0.399	**−0.939**
Gd	–0.654	–0.702	–0.673	–0.799	–0.678	–0.642	–0.668	–0.669	**−0.835**	–0.769	–0.650	–0.592	0.767	–0.643
Tl	–0.200	–0.299	–0.378	–0.398	–0.449	–0.374	–0.375	–0.321	–0.539	–0.393	–0.511	–0.079	–0.022	–0.562
Bi	–0.200	–0.299	–0.378	–0.398	–0.449	–0.374	–0.375	–0.321	–0.539	–0.393	–0.511	–0.079	–0.022	–0.562

In Table 7 was presented the correlations between the selected fungi genera and the PAHs content. All correlations between fungi and PAHs were presented in the Supplementary Materials – Table 2. Strong positive correlations were confirmed between: *Alternaria* and Fluoranthene (*r* = 0,874), Pyrene (*r* = 0,834) and Chrysene (*r* = 0,823) and also between *Boeremia* and Acenaphthylene (*r* = 0.825) and Acenaphthene (*r* = 0.830). The strong positive correlation was confirm also between *Alternaria*, *Knufia*, *Cladophialophora*, *Cystobasidium*, *Scolecobasidium*, *Didymella*, and Pyrene, Chrysene and Benz[a]anthracene. Also strong correlation was presented between *Tetracladium*, *Symbiotaphrina*, *Exophiala*, and Acenaphthylene, Acenaphthene, Fluorene. Benzo[a]pyrene content was correlated with *Boeremia*, *Tetracladium*, *Symbiotaphrina*, *Exophiala*, and *Cystobasidium*. The strong correlations between fungi genera and PAHs may indicate a high bioremediation potential of fungi.

**TABLE 7:** Pearson’s correlation coefficient (*P* ≤ 0.05) of selected fungi genera and PAHs (data marked in bold are statistically significant).

	*Alternaria*	*Knufia*	*Clonostachys*	*Boeremia*	*Septoriella*	*Tetracladium*	*Symbiotaphrina*	*Exophiala*	*Cladophialophora*	*Cystobasidium*	*Scolecobasidium*	*Phallus*	*Didymella*	*Devriesia*
Naphthalene	–0.184	0.138	**−0.816**	0.161	0.037	0.498	0.089	0.319	0.147	0.173	0.132	**0.920**	0.241	0.520
Acenaphthylene	–0.043	0.555	–0.771	**0.825**	0.537	**0.937**	**0.855**	**0.965**	0.711	0.786	0.705	0.665	0.756	**0.813**
Acenaphthene	–0.016	0.569	–0.782	**0.830**	0.553	**0.931**	**0.854**	**0.970**	0.719	0.794	0.713	0.658	0.763	0.811
Fluorene	–0.029	0.562	**−0.822**	0.806	0.534	**0.935**	**0.828**	**0.955**	0.702	0.776	0.695	0.714	0.753	**0.818**
Anthracene	0.341	0.607	**−0.975**	0.591	0.530	0.638	0.485	0.682	0.526	0.571	0.515	0.688	0.580	0.614
Phenanthrene	0.241	0.507	**−0.965**	0.491	0.230	0.623	0.185	0.482	0.326	0.571	0.215	0.658	0.540	0.611
Fluoranthene	**0.874**	0.719	–0.486	0.440	0.721	0.120	0.389	0.439	0.517	0.522	0.518	–0.105	0.488	0.102
Pyrene	**0.834**	**0.933**	–0.579	0.514	**0.934**	0.302	0.695	0.689	**0.824**	**0.824**	**0.825**	0.046	0.805	0.172
Chrysene	**0.823**	**0.960**	–0.599	0.449	**0.937**	0.287	0.694	0.664	**0.840**	**0.830**	**0.838**	0.108	**0.828**	0.130
Benz[a] anthracene	0.798	**0.972**	–0.611	0.462	**0.949**	0.319	0.728	0.694	**0.867**	**0.858**	**0.866**	0.136	**0.858**	0.150
Benzo[b] fluoranthene	0.729	**0.830**	–0.433	0.233	0.689	0.209	0.465	0.286	0.349	0.461	0.447	0.215	0.620	0.202
Benzo[k] fluoranthene	0.789	**0.830**	–0.633	0.533	0.789	0.309	0.565	0.586	0.649	0.661	0.647	0.115	0.640	0.222
Benzo[a]pyrene	0.183	0.677	**−0.824**	**0.880**	0.660	**0.888**	**0.870**	**0.986**	0.761	**0,834**	0.756	0.571	0.794	0.776
Indeno[1,2,3-cd] pyrene	0.053	0.619	–0.795	**0.850**	0.600	**0.922**	**0.876**	**0.983**	0.746	**0,820**	0.740	0.630	0.787	0.796

**FIGURE 7:**
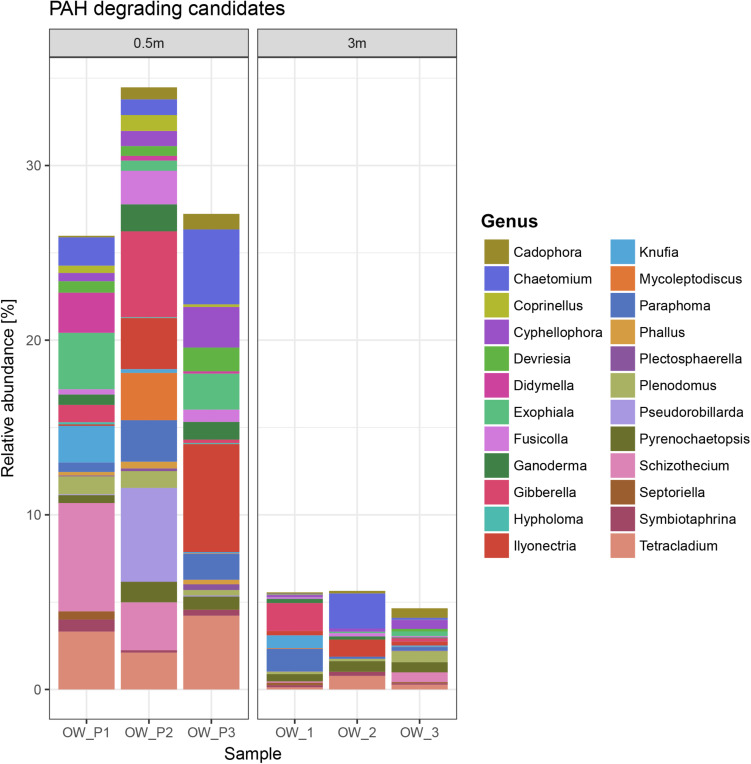
PAH-degrading candidates.

The mycobiome of the soils collected directly from the oil wells (OWP1, OWP2, and OWP3) had a 35% share of PAH-degrading candidates, compared to the soil collected 3 m from the oil wells (OW1, OW2, and OW3), which was < 5% (Figure 7). The main candidates for PAH-degrading fungi belong to genera *Ilyonectria, Chaetomium, Gibberella*, *Paraphoma*, *Schizothecium*, *Pseudorobillarda*, *Tetracladium*, *Ganoderma*, *Cadophora*, *Exophiala*, *Knufia*, *Mycoleptodiscus*, *Cyphellophora*, *Fusicolla*, *Devriesia*, *Didymella, Plenodomus, Pyrenochaetopsis, Symbiotaphrina, Phallus, Coprinellus, Plectosphaerella*, *Septoriella, and Hypholoma*.

## Discussion

The results of the current study focused on differences in both structural and metabolic fungal biodiversity in crude-oil-contaminated soils after long-term natural bioremediation. According to the literature, long-term soil contamination with crude oil leads to the adaptation of microorganisms that live there (including fungi) and, consequently, the creation of a number of adaptation mechanisms (Chen et al., 2006; Gałązka et al., 2018; Grządziel and Gałązka, 2019). Additionally, during long-term contamination the soil is richly populated by ruderal vegetation, which spontaneously inhabit these areas. The rhizosphere of these plants is a very interesting and diverse habitat of soil microorganisms that are capable of growing in contamination conditions (Adenipekun and Lawal, 2012; Alori et al., 2017).

The measurement of community level physiological profiles and soil microbial communities can be used as indications of the biological activities and natural biochemical processes in contaminated soil (Frąc et al., 2018). The Biolog FFPlates are widely accepted as a sensitive tool to indicate viable microbes and provide an activity fingerprint for the fungal community composition. The numbers of different groups of fungi are also an indicator of changes taking place in the contaminated soil environment (Gałązka and Gałązka, 2015). In our study, high activity in the metabolic profile was demonstrated in soil samples taken directly from under the oil well (OWP1, OWP3). These data may indicate higher activity and biodiversity in the soil. Many studies include higher fungal diversity in contaminated soils compared to control soils (Winquist et al., 2014, Wolińska et al., 2018; Deshmukh et al., 2016; Borowik et al., 2019). Fungi adapt very quickly to existing conditions and are able to metabolize contaminants in a biological way. In our research, we showed that control soils (3 m away from the oil well) which were also covered with meadow vegetation, were characterized by much higher biodiversity. However, a higher percentage of fungi capable of decomposing PAHs was found in the soil OWP (from the oil well) that was not covered by vegetation. Hence, it should be assumed that vegetation is largely responsible for the increase in biodiversity and microbial activity (Gałązka and Grządziel, 2018; Zhang et al., 2019; Lin et al., 2020). On the other hand, it may be so that the components of the crude oil which are toxic to fungal taxa at those sites, therefore less fungal diversity and activity, but higher abundance of PAH-degraders which are acclimated to the contamination. This is confirmed in our study by the results of the correlation of PAHs and trace elements with the number of fungi at the level of the parent. In study Blasi et al. (2016) were found in contaminated soils *Exophiala mesophila* and *Cladophialophora immunda*. Also in our research a large percentage of *Exophiala* spp. was also found in the group of PAH-degrading fungi, which were not found in the soils collected within the 3 m radius of the oil wells.

The mycobiome of the soils collected directly from the oil wells (OWP1, OWP2, and OWP3) had a higher share of fungi that potentially have the ability to utilize PAHs compared to the soil collected 3 m from the oil wells (OW1, OW2, and OW3). The main candidates belong to genera *Ilyonectria, Chaetomium, Gibberella, Paraphoma, Schizothecium, Pseudorobillarda, Exophiala, Knufia, Mycoleptodiscus, Cyphellophora, Fusicolla, Devriesia, Didymella, Phallus, Coprinellus*, and *Hypholoma*. Many authors confirm that fungi with bioremediation potential were isolated from soils contaminated with petroleum substances (Steffen et al., 2002; Prenafeta-Boldú et al., 2006; Huang et al., 2007; Gao et al., 2010; Yuan et al., 2010; Lin et al., 2020). For example *Exophiala macquariensis* with very high bioremediation potential was identified in a hydrocarbon contaminated sub-Antarctic soil (Zhang et al., 2019). Also the presence of Schizothecium was found in historically oil-contaminated soil (Covino et al., 2016). Also in our research, these two genera (*Exophiala* and *Schizothecium*) were found in contaminated soils (OWP1, OWP2, and OWP3).

The share of three- and four-ringed PAHs was higher as the distance from the oil well increased (except for location 1, where the three-ringed PAH contribution was 1.5-times higher close to the oil well). This finding indicates that more effective degradation processes occur closer to the oil wells.

Bioaugmentation, cometabolism, and ryzodegradation processes are well known bioremediation methods used to remove organic compounds from soils. These processes are increasingly supported by genetic engineering and molecular biology techniques (Prenafeta-Boldú et al., 2006; Gałązka et al., 2018). Further, toxicological data and soil physicochemical characterization should be closely interconnected in order to make adequate and informed decisions. The ecotoxicological component should be permanently placed in the scheme of modern environmental management, and the appropriate risk assessment (ERA) should be performed in accordance with the recommendations of the European Union and the US Environmental Protection Agency (US-EPA, 1997).

Sites contaminated by recalcitrant organic compounds have often been shown to be characterized by the concomitant presence of trace elements. Bhattacharya et al. (2014) shown that *Pleurotus ostreatus* can utilize benzo(a)pyrene while using heavy metal cations and vanillin and 2,2- azinobis-(3-ethylbenzothiazoline-6-sulfonate) as mediators in bioremediation. This process enhance the degradation by ∼74.2%.

Also in our studies, increased amounts of trace elements were found in contaminated soil. Nevertheless, these trace elements can be of great importance in the process of growth and development of particular fungi in contaminated site. These elements can be of great importance in the selection of fungi to use PAHs as the sole carbon and energy source. In study of D’Annibale et al. (2006) in historically polluted soils were found fungi isolated from an aged and heavily contaminated soil. Among them, *Allescheriella* sp., *Stachybotrys* sp., and *Phlebia* sp. were selected to assess their degradative potential. Bioremediation in the presence of fungi *Aspergillus restrictus*, *Chrysosporium keratinophilum*, *Fusarium solani*, *Gliocladium roseum*, *Penicillium*, and *Stemphylium* exhibited substantial removal of petroleum hydrocarbons from soil contaminated with petrol and diesel at short incubation periods as indicated by enhanced total organic carbon (TOC) removal (Karigar and Rao, 2011; Maruthi et al., 2013). Additionally, some trace elements can also be bound by carbonates and oxalates produced by fungi. The functional groups located on the surface of the cell wall of fungi are also extremely important in the binding of trace elements, especially those trace elements that are often overlooked and do not receive much research interest (e.g., beryllium, vanadium, antimony, cerium, and gadolinium).

In our research, we showed that soils OW which were also covered with meadow vegetation, were characterized by much higher by quantity glomalin-related soil proteins content compared to OWP samples (without plants). The higher number of AM fungi was observed exactly in soil samples collected from 3 m away from the oil well. Additionally, at 3 m from the oil wells, there was significant vegetation: plantain (*Plantago major* L.), field clover (*Trifolium arvense* L.), common dandelion (*Taraxacum officinale* FH Wiggers coll.), common starweed (*Stellaria* media L.) and fescue (*Festuca pratensis* L.), among others. With these plants, mycorrhizal fungi establish a close symbiosis and thus find suitable conditions for glycoprotein production. Higher GRSP content in soil samples from 3 m of the oil (OW1, OW2, and OW3) may indicate that the soil subjected to a long-term natural remediation may already have sufficient conditions for the growth and development of mycorrhizal fungi. In these samples, where remediation had occurred for many years, there was already a natural remediation, denoted by marked variety and variability of the sequenced classes.

AM fungi produced GRSP participate in the bioremediation process both directly (by affecting the pathways of degradation of PAHs) and indirectly (influence on the soil structure, oxygenation and the supply of carbon, regulation of the ratio C to N), (Rodriguez et al., 2009; Nwaichi et al., 2015). GRSP production and its role in soil structure stabilization becomes an alternative method of supporting bioremediation biological processes for soils contaminated with PAHs and heavy metals (Ciesielczuk et al., 2014). Mycorrhiza also proved to be particularly useful in phytostabilization processes (Gonzalez-Chavez et al., 2004).

The processes of biological contaminant removal from contaminated areas mainly use saprobiotic fungi, while the role of mycorrhizal fungi is still unknown and likely underestimated. Meanwhile, properly developed mycorrhiza may increase plant survival in difficult conditions by increasing the availability of nutrients, reducing the stress associated with low water availability, increasing phytohormone production and improving the structure of the substrate, all of which can significantly increase the effectiveness of bioremediation (Blasi et al., 2016).

Long-term oil-contaminated soil is low in nitrogen and phosphorus compounds and has low water retention capacity and notable susceptibility to wind erosion. The spontaneous colonization of such a medium by arbuscular fungi is a long-term process. Mycelium of some species, such as *Glomus mosseae* that tolerate the presence of elevated metal concentrations, can bind several times greater amounts of metals (about 65–75%) than the mycelium of the species often used in bioremediation, namely *Rhizopus orrhizus* (35–45%; Khan, 2005).

The ability to bind and detoxify metals in the underground parts of the plant, obtained through mycorrhiza, is particularly useful for stimulating crop plant growth on contaminated substrates. These plants are grafted with a mycelium isolated from the metallophyte *Viola calaminaria*. Strains isolated from contaminated sites are much more useful than strains that originate from non-contaminated sites. The strain derived from *V. calaminaria* has the highest metal binding efficiency among the tested strains (73%) (Joner et al., 2007).

Mycorrhizal fungi, through GRSP production, significantly increase the efficiency of the mycoremediation process. They reduce the spread of metals to other areas via their penetration into the soil profile and groundwater. In one study, 0.08 mg Cd, 4.3 mg Cu and 1.12 mg Pb was extracted from laboratory plants inoculated with mycorrhizal fungus from 1 g glomalin. An *in vitro* experiment showed that *Gigaspora rosea* sequestered 28 mg Cu per g glomalin, which constituted 35% of the total Cu added to the substrate (Ciesielczuk et al., 2014).

In our research, soil taken directly from the extract of crude oil was characterized by a higher number of PAH degrading fungi. Soil fungi can adapt to adverse conditions, e.g., organic pollution, in a very short time. So far, however, the mechanisms of how plant pathogens can adapt to sites of confluence and whether they can use PAHs as the only source of carbon and energy have not been explained.

Although the first plants that colonize areas with an increased content of petroleum-derived substances and trace elements are usually non-mycorrhizal species, the education of a dense plant cover, as well as the improvement of soil structure, depends on the appearance of symbiotic fungi. This phenomenon is particularly important in habitats such as areas contaminated with post-flotation material and refinery locations.

## Conclusion

1. The results of the current study indicated significant differences in both structural and metabolic fungal biodiversity in crude-oil-contaminated soils after long-term natural bioremediation.
2. The soil samples collected directly from oil well had higher biodiversity values compared to soils collected 3 m from the wells. Further, these soils had different structural diversity and different groups of some fungi compared to the soil taken 3 m from the oil wells.
3. The mycobiome of the soils collected directly from the oil wells (OWP1, OWP2, and OWP3) was characterized by a 35% share of PAH-degrading candidates, compared to the soil collected at the 3 m distance from the oil wells (OW1, OW2, and OW3), which was < 5%. Strong correlations between fungi genera and PAHs content was confirmed.
4. Naturally occurring bioremediation processes at the contamination site is highly important due to the possibility of recruitment of autochthonous fungal strains capable of decomposing pollutants.

## Data Availability Statement

The datasets generated for this study can be found in online repositories. The names of the repository/repositories and accession number(s) can be found in the article/Supplementary Material.

## Author Contributions

AG conceived and designed the experiment. All the other authors participated in writing, statistical, graphical elaboration, and manuscript revision, provided final approval of the manuscript and agreed to be accountable for all aspects of the work.

## Conflict of Interest

The authors declare that the research was conducted in the absence of any commercial or financial relationships that could be construed as a potential conflict of interest.
